# Supplementary material for: Evaluation of Anti-Hyperglycemia and Complications of Red and Black Thai Jasmine Rice Cultivars in Streptozotocin-Induced Diabetic Rats
Source: Molecules. 2022 Nov 19;27(22):8043. doi: 10.3390/molecules27228043 (PMC9699537; doi:10.3390/molecules27228043)
Supplement: Supplementary file 1 [file molecules-27-08043-s001.zip › molecules-2004753-supplementary.pdf]

Materials and methods (Supplementary Materials)

1.1 Quantitative analysis of RR and BR extracts

1.1.1 Phenolic compound analysis

HPLC separation was performed on a LiChroCART RP-18e column [150 x 4.6 mm, diameter 5 µm] (Purospher STAR Merck USA). The mobile phase consisted of 10 mM ammonium formate buffer, pH 4 with formic acid (mobile phase A) and 10 mM ammonium formate buffer, pH 4 with formic acid in acetonitrile (m(mobile phase B)). The gradient used was 100% of solvent B from 0 - 5 min, 0% to20% of solvent A from 5 - 10 min, 20% of solvent A from 10-20 min, and 20% to 40% of solvent A from 20-60 min, at flow rate of 1.0 mL/min and the injection volume was 10 µL. The separated compounds, acquired with a diode array at wavelengths of 270 nm, were assessed and compared to a standard calibration curve.

1.1.2 Anthocyanin analysis

HPLC separation was performed on a Zorbax SB C18 column [150 x 4.6 mm, diameter 5 µm] (Agilent technologies USA)). The mobile phase was composed of 0.1% trifluoroacetic acid (TFA)) in water (mobile phase A) and 0.1% TFA in acetonitrile (mobile phase B). The gradient used was 7% to 20% of solvent A in 60 min, then 93% to 80% of solvent B in 60 min at flow rate of 0.5 mL/min and the injection volume was 10 µL. Separated compounds, acquired with a diode array at wavelengths of 270 and 500 nm, were interpreted and quantified using a standard calibration curve.

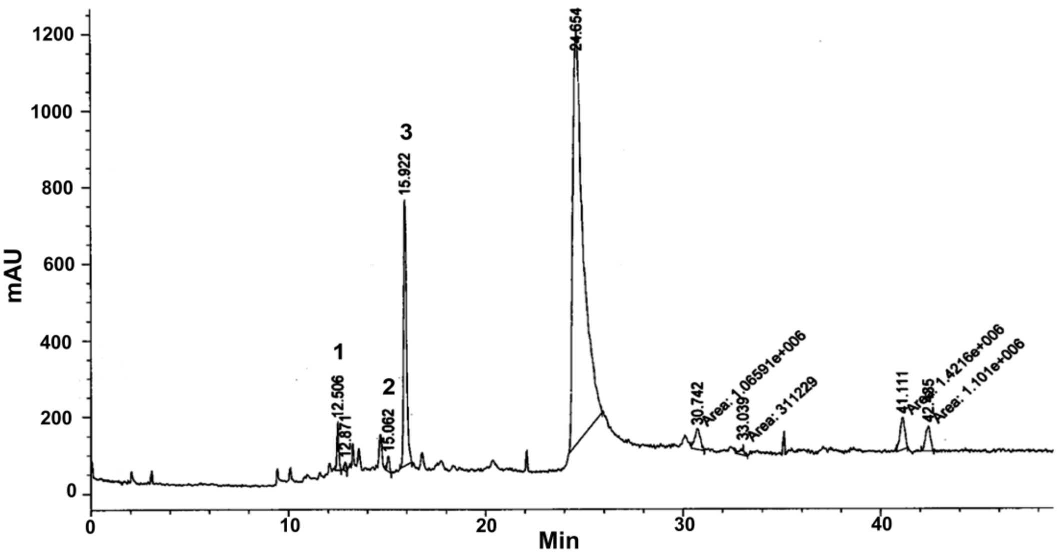

Figure S1 High performance liquid chromatography (HPLC) chromatogram of a mixture of phenolic acid standards. Peak: 1 Catechin, 2 Rutin, and 3 Isoquecetin.

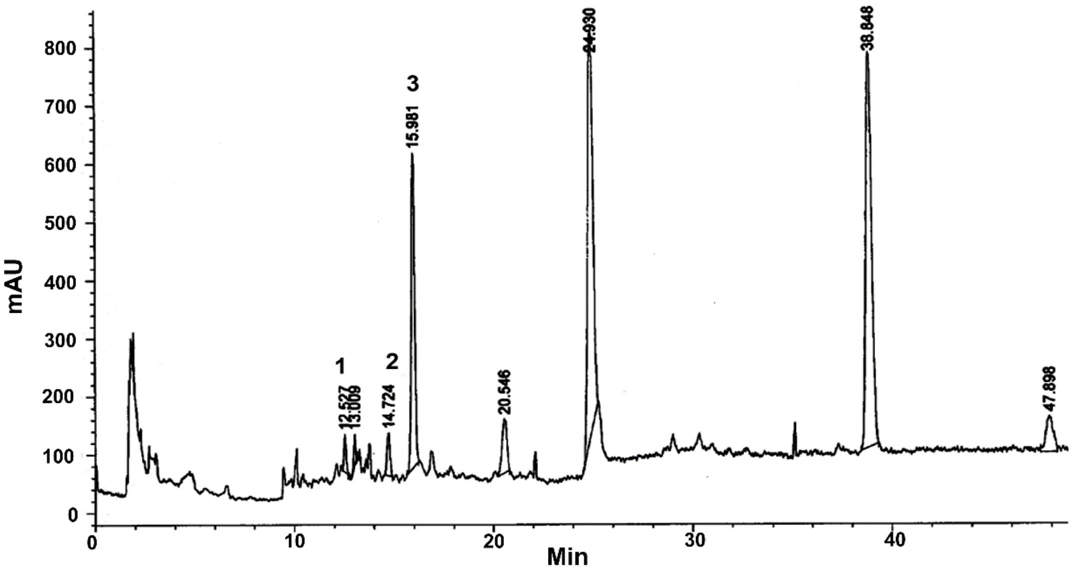

Figure S2 HPLC chromatogram of polyphenols from red rice extract. Peak: 1 Catechin, 2 Rutin, and 3 Isoquecetin.

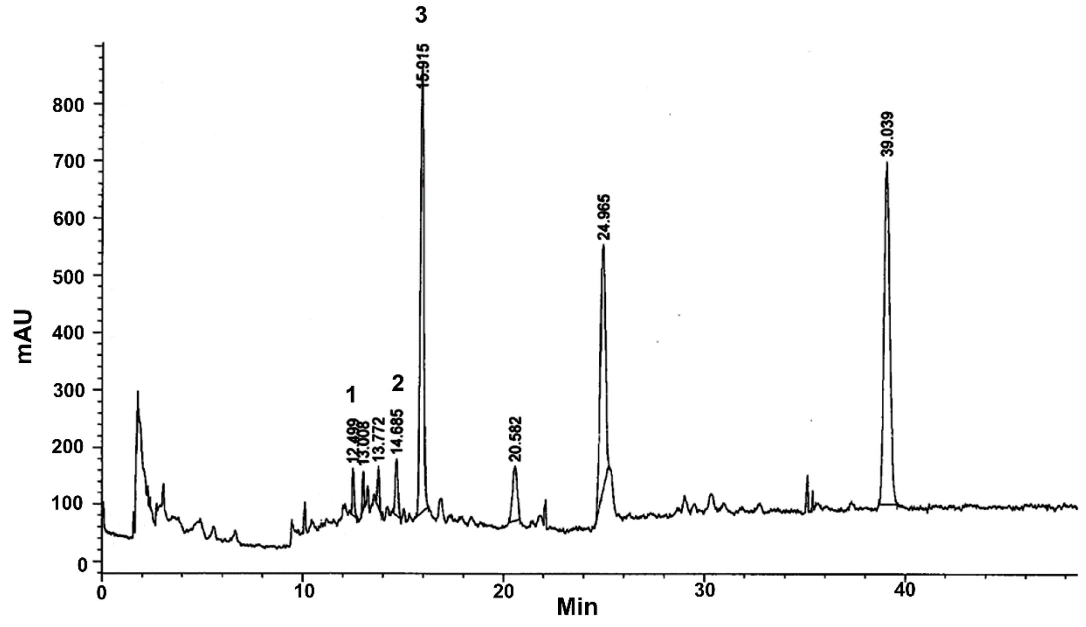

Figure S3 HPLC chromatogram of polyphenols from black rice extract. Peak: 1 Catechin, 2 Rutin, and 3 Isoquecetin.

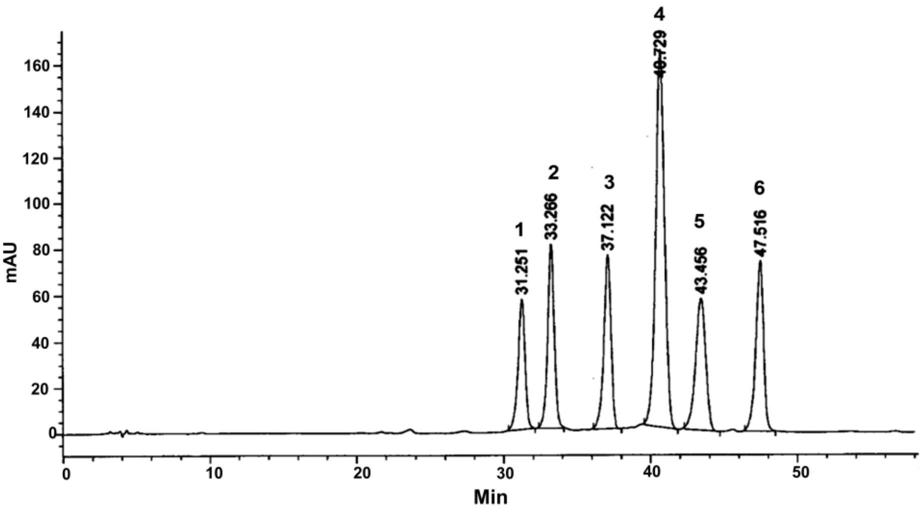

Figure S4 HPLC chromatogram of standard anthocyanins. Peak: 1 cyanidin 3-glucoside, 2 cyanidin 3-O-rutinoside, 3 Callistephin, 4 Peonidin, 5 Malvidin, and 6 Quercetin.

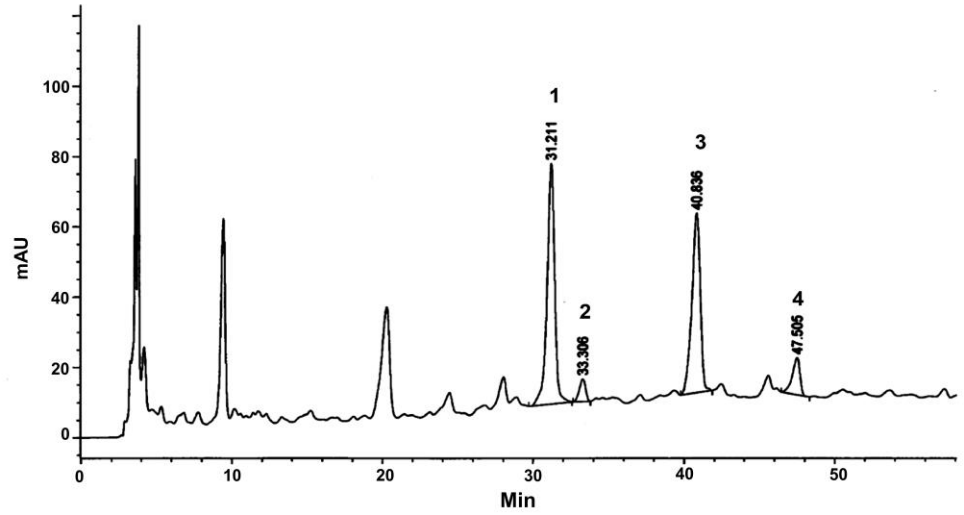

Figure S5 HPLC chromatogram of anthocyanins from red rice extract. Peak: 1 cyanidin 3-glucoside, 2 cyanidin 3-O-rutinoside, 3 Peonidin, and 4 Quercetin

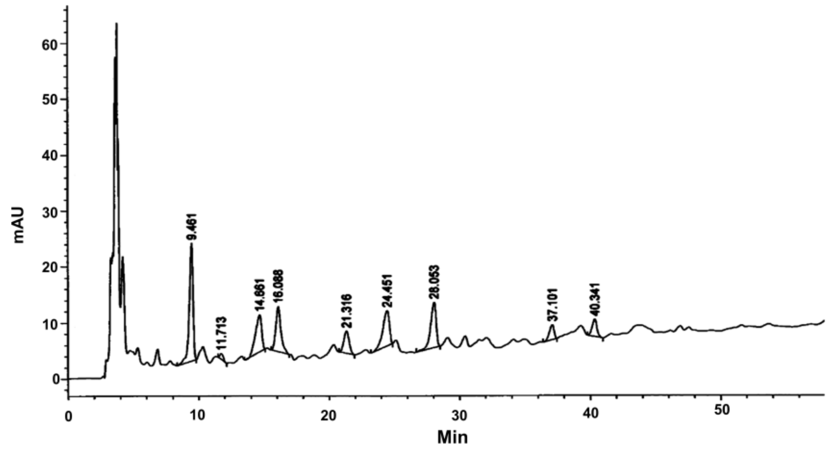

Figure S6 HPLC chromatogram of anthocyanins from black rice extract.

Table S1. Effect of RR and BR on body weight, blood glucose, triglyceride, cholesterol, AST, ALT, BUN and creatinine levels in normal control rats.

Group	D0	D15	D30	D45
**Body weight (g)**				
Normal RR treated group	276.25 ± 4.89	296.67 ± 3.65	335.83 ± 4.80	404.17 ± 5.29
Normal BR treated group	266.25 ± 3.50	291.88 ± 2.98	323.13 ± 2.82	396.25 ± 4.09
**Blood glucose level (mg/dL))**				
Normal RR treated group	152.37 ± 11.34	213.02 ± 6.31	207.74 ± 8.25	229.52 ± 23.57
Normal BR treated group	158.50 ± 8.78	208.67 ± 4.15	214.48 ± 9.83	236.25 ± 31.53
**Triglyceride level (mg/dL)**				
Normal RR treated group	118.69 ± 2.88	121.88 ± 6.55	120.25 ± 11.55	130.07 ± 3.74
Normal BR treated group	110.81 ± 2.02	118.44 ± 3.04	115.25 ± 2.92	121.18 ± 4.43
**Cholesterol level (mg/dL)**				
Normal RR treated group	156.63 ± 7.67	175.00 ± 8.44	193.20 ± 6.21	191.36 ± 5.08
Normal BR treated group	144.75 ± 9.92	125.25 ± 7.59	155.13 ± 9.34	170.39 ± 7.19
**AST ((Unit/L)**				
Normal RR treated group	59.05 ± 2.98	59.23 ± 1.59	60.28 ± 1.52	62.28 ± 1.13
Normal BR treated group	60.84 ± 2.06	58.07 ± 5.40	68.76 ± 3.52	69.58 ± 4.61
**ALT (Unit/L)**				
Normal RR treated group	54.34 ± 2.06	56.79 ± 2.14	62.67 ± 3.43	71.67 ± 3.46
Normal BR treated group	54.53 ± 2.67	50.21 ± 3.87	64.11 ± 4.51	63.71 ± 3.56
**BUN (mg/Dl)**				
Normal RR treated group	0.85 ± 0.15	0.88 ± 0.19	1.02 ± 0.04	0.98 ± 0.09
Normal BR treated group	0.91 ± 0.07	1.13 ± 0.04	0.91 ± 0.02	0.81 ± 0.04
**Creatinine (mg/Dl)**				

Normal RR treated group	0.382 ± 0.02	0.440 ± 0.06	0.455 ± 0.03	0.492 ± 0.10
Normal BR treated group	0.331 ± 0.05	0.423 ± 0.03	0.486 ± 0.02	0.538 ± 0.10

Table S2 Effect of RR and BR on MDA, FRAP, GSH in serum and GSH in liver tissue in normal control rats.

Group	MDA (μmol/dL)	FRAP (μmol FeSO4/L)	GSH (nmol GSH/μg protein) in Serum	GSH (nmol GSH/μg protein) in liver tissue
Normal RR treated group	0.497 ± 0.10	1942.92 ± 71.67	12.34 ± 1.87	8.54 ± 0.72
Normal BR treated group	0.495 ± 0.12	1849.69 ±1 01.46	8.93 ± 1.10	6.7 ± 2.05
